# Capecitabine and mitomycin C in patients with metastatic colorectal cancer resistant to fluorouracil and irinotecan

**DOI:** 10.1038/sj.bjc.6603021

**Published:** 2006-02-21

**Authors:** C Alliot

**Affiliations:** 1 Hematology/Oncology Division, General Hospital of Annemasse, BP525, 74107 Annemasse Cedex, France

**Sir**,

In the 5 September issue, Chong *et al* (2005) reported a phase II study of 36 patients with metastatic colorectal cancer treated with a third-line chemotherapy consisting of capecitabine and mitomycin. The response rate was 15.2%, median failure-free survival, 5.4 months, and median overall survival, 9.3 months. No grade 4 toxicity was observed. Although this regimen seems particularly convenient, the study is questionable by several aspects. The first point is the imprecision regarding prognostic factors. In particular, the number of metastatic sites and several biological factors such as albumin, lactate dehydrogenase, alkaline phosphatase or carcinoembryonic antigen have not been detailed. Moreover, there is no mention about the proportion of patients who have been operated for a primary tumour. Since these patients benefit from a follow-up, early detection of metastases can be favoured, leading to the selection of patients with low tumour burden and finally, to a potential advantage in terms of therapeutic efficacy and tolerance. Another point is the first-line therapy which consisted only of 5-fluorouracil (5-FU) in 33 patients, although only two have had received oxaliplatin. Moreover, there is no precision regarding the type of 5-FU-based regimen since a longer progression-free survival has been clearly demonstrated with the LV5FU2 regimen (bimonthly combination of high-dose leucovorin and 5-FU bolus plus continuous infusion) comparatively with the monthly schedule of low-dose leucovorin and 5-FU (de Gramont *et al*, 1997). The authors compare favourably this regimen with results of third-line treatments in randomised phase III studies, while it can be admitted that the patients accrued in phase II studies inevitably are more selected. Another indirect comparison with cetuximab is questionable since targeted therapies offer their best activity in combination with chemotherapy (Cunningham *et al*, 2004). Conceptually, the probability of response of mitomycin-C after either oxaliplatin or irinotecan is extremely low. Moreover, probably oral fluoropyrimidines will replace 5-FU in a vast proportion of the first-line combinations in advanced diseases, and probably in the adjuvant setting (Twelves *et al*, 2005). The efficacy of capecitabine in second-line combinations also might be discussed as illustrated by several recent phase II studies. No response has been obtained in 22 patients of the MD Anderson Cancer Center with 5-FU-resistance (Hoff *et al*, 2004), and in 51 Korean patients refractory to 5-FU and leucovorin (Lee *et al*, 2004). The same inefficacy has been observed in 20 Swedish patients who all had received 5-FU, irinotecan and oxaliplatin (Gubanski *et al*, 2005). Finally, a poor response rate of 4.8% has been obtained in 21 Korean patients receiving mitomycin-C and capecitabine as third-line chemotherapy after various combinations of 5-FU, irinotecan and oxaliplatin (Lim *et al*, 2005). Although toxicity seems moderate, grade 2 stomatitis or grade 2 diarrhoea may deteriorate quality of life. Moreover, patients participating to therapeutic trials in university centres are particularly well followed. In this line, the lack of haemolytic uraemic syndrome should not lead to excessive self-confidence. In conclusion, this trial does not reflect the current practice in which irinotecan, oxaliplatin and targeted therapies are integrated into the two first therapeutic lines. Nevertheless, mitomycin might eventually be tested in patients ineligible for reference drugs.
